# Effects of different opioids on the contractile tension of isolated rat intestinal smooth muscle

**DOI:** 10.3389/fphar.2025.1617936

**Published:** 2025-10-27

**Authors:** Chen Yang, Jianying Hu, Yan Cheng, Shen Sun, Shaoqiang Huang

**Affiliations:** ^1^ Department of Anesthesiology, Obstetrics & Gynecology Hospital, Fudan University, Shanghai, China; ^2^ Department of Anesthesiology, Shanghai Fifth People’s Hospital, Fudan University, Shanghai, China

**Keywords:** opioids, contractile tension, contractile frequency, intestinal smooth muscle, rat

## Abstract

**Backgroud:**

To compare how five common opioids affect rat intestinal muscle tension and frequency across concentrations, establishing an animal model to provide translational evidence for selecting opioids with the least gastrointestinal impact.

**Methods:**

Isolated rat small intestine specimens were prepared, and six intestinal segments were selected from the same rat and placed into remifentanil, sufentanil, oxycodone, nalbuphine, butorphanol, and the control group with balanced solution. Experiments were carried out to observe and compare the changes in the contraction tension and contraction frequency of small intestinal smooth muscle during the change in concentration gradients of 10–^9^, 10–^8^, 10–^7^, 10–^6^, 10–^5^, and 10–^4^ M. Statistical analysis obtained from 10 rats in the *in vitro* intestinal tube experiment was performed.

**Results:**

The results revealed that when the concentration increased to 10^−7^ M, the intestinal contraction tension in the butorphanol group began to decrease significantly, and the intestinal contraction tension in the nalbuphine and oxycodone groups began to decrease significantly at concentrations of 10^−6^ M. while the sufentanil group and the remifentanil group showed a significant decrease in intestinal contractile tension at 10^−5^ M and 10^−4^ M, respectively. When the concentration of the drug increased to 10^−4^ M, the contraction frequency of the sufentanil group decreased significantly, and there was no statistical difference among concentrations of other drugs.

**Conclusion:**

As the concentration increased, different opioids inhibited the contractile tone of rat intestinal smooth muscle *in vitro*. The intensity of inhibition was butorphanol > nalbuphine ≈ oxycodone > sufentanil > remifentanil, and the Contraction frequency was almost unaffected except in the very high concentration sufentanil group.

## Introduction

Postoperative gastrointestinal dysfunction (PGID) is a common adverse reaction. Its pathogenesis is complex and affected by multiple factors (Mythen, 2009). Opioids used for anesthesia and analgesia are one of them (Imam et al., 2018; Müller-Lissner et al., 2017). Many animal experiments have confirmed morphine and sufentanil may dose dependently increase the contractile tension and contraction ability of isolated rat small intestine smooth muscle, while dezocine has no significant effect on intestine smooth muscle contraction (Bian et al., 2015). Clinical studies have also found that the incidence of postoperative nausea and vomiting is lower in patients receiving intravenous oxycodone compared with sufentanil during laparoscopic surgery (Tao et al., 2019). However, the comparison between drugs has not been made in most cases, even if the mechanism of drugs is more clear, it can`t play a guiding role in clinical medication selection of opioids. There are many options for opioids during anesthesia and analgesia: MOP receptor agonists (sufentanil, remifentanil), KOP receptor agonists (nalbuphine, butorphanol), MOP and KOP dual receptor agonists (oxycodone), which act on different receptors, and the clinical doses are also different (Kvam et al., 2004; Gepts et al., 1995; Egan, 1998; He et al., 2021; Opioids for pain, 2022; Raff et al., 2019). It is not clear which of these drugs has the least and the most severe inhibition of gastrointestinal function. It is possible to find the drug with the least inhibitory effect on gastrointestinal function through studies comparing several different drugs simultaneously under the same conditions. In this way, on the premise of meeting the requirements of analgesia, drugs with less inhibition on gastrointestinal function can be preferred clinically.

Therefore, we designed this *in vitro* animal experiment to compare the effects of various opioids on the motility of isolated rat intestinal smooth muscle at different concentrations.

## Methods

### Laboratory animal

All animal experiments were performed in accordance with the basic principles of Fudan University’s animal experiments. The Animal Ethics Committee of Shanghai Medical College of Fudan University approved the protocol (201907007Z), and specific-pathogen-free (SPF) 10 Sprague Dawley rats, half male and half female, weighing between 200–220 g, were purchased from Shanghai Jiesi Laboratory Animal Co., Ltd. Before the experiment, they were placed in standard cages and reared for 1 week in a 12 h:12 h light-dark cycle, with food and water *ad libitum*.

### Experimental process

Rats were fasted for 10 h before the experiment and had free access to water. On the day of the experiment, all rats were euthanized by inhaling carbon dioxide (replacement rate30%-70%). After the rat was determined to have died, the peritoneal cavity was exposed through a median incision. Identify the ligament of Treitz, isolate the segment from jejunum to ileum, trim away the mesentery along the intestinal wall, and rinse the segment in a Petri dish. Physiological saline solution (PSS) was pre-prepared (8.0 g of NaCl, 0.2 g of KCl, 0.214 g of MgCl_2_ 6H_2_O, 0.05 g of NaH_2_PO_4_, 1.0 g of NaHCo_3_, 0.2 g of CaCl_2_, 1.0 g of glucose, and 1,000 mL of double distilled water). The intestinal contents were rinsed with a 10 mL syringe in PPS, and the intestinal segments were cut into 1 cm small segments (at least six segments per rat).

One end of the intestinal tube was connected with a tension transducer (JZJ01 type, China Chengdu instrument Factory) with cotton thread, and the other end of the intestinal tube was fixed in the smooth muscle groove with an “L”-shaped hook. The intestinal segment was completely immersed in a smooth muscle tank filled with 30 mL of Tyrode’s solution, and the temperature in the bath was kept constant at 37 °C. The smooth muscle tank was inflated with an oxygen pump (95% O2, 5% CO2 ventilation, two air bubbles/s). The connection between the intestinal tube and the tension transducer was adjusted so that it had a certain degree of tension, and the preload was set to 1 g.

**FIGURE 1:**
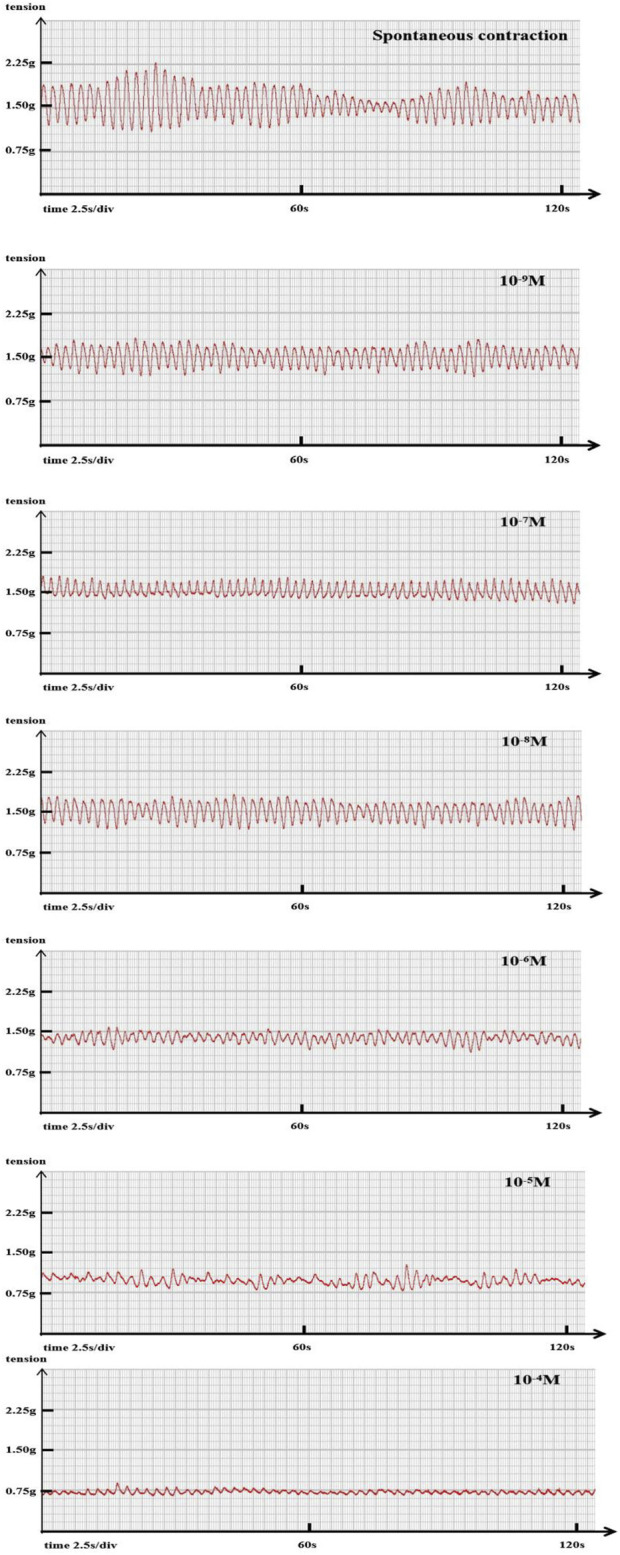
Representative original trace A representative example showing the changes in ileal segment contraction tension and frequency with increasing sufentanil concentration. Baseline: tension 1.15 g, frequency 27 cycles/min. Sufentanil 10^−9^ M: tension 0.89 g, frequency 24 cycles/min; 10^−8^ M: 0.77 g, 17 cycles/min; 10^−7^ M: 0.71 g, 17 cycles/min; 10^−6^ M: 0.67 g, 14 cycles/min; 10^−5^ M: 0.52 g, 14 cycles/min; 10^−4^ M: 0.34 g, 10 cycles/min.

The tension transducer is connected to a polyconductive physiological recorder (RM6240BD, China Chengdu Instrument Factory) to record the signal changes of isometric contraction of jejunal smooth muscle. The processed signal of the RM6240 (USB-3.8 version) biological signal acquisition and processing system is displayed on the computer display scree (Figure 1).

n. Six intestinal segments from the same rat were selected, placed in six constant temperature water baths, and randomly divided into six groups for synchronous experiments. Sufentanil citrate, remifentanil hydrochloride, oxycodone hydrochloride, nalbuphine hydrochloride, butorphanol tartrate, and PSS were added respectively in stepwise increasing concentrations.The experimental liquid was calculated and prepared according to the molecular weight of the drug. At 10^−4^ M, remifentanil is 41.29 μg/mL, and sufentanil is 57.87 μg/mL oxycodone 35.18 μg/mL, nalbuphine 42.09 μg/mL, butorphanol 47.76 μg/mL, and configuration concentration gradients of 10^−9^, 10^−8^, 10^−7^, 10^−6^, 10^−5,^ and 10^−4^ M were diluted with PSS.

The small intestine segments mounted longitudinally equilibrated for 30 min, and after the spontaneous contraction activity was stable, the contraction tension and contraction frequency were observed, and recorded as baseline values. Then, the experiment proceeded with stepwise increases in concentration from low to high, without washout between concentrations. Each concentration was studied for 10 min, the intestinal segment was balanced at this concentration for the first 5min, and the intestinal muscle contraction frequency and contraction tension were recorded for the next 5 min. During the whole experiment, the control group synchronously replaced the same PSS and the contraction tension, contraction frequency was recorded, the total contraction time remained consistent. In order to exclude individual differences of intestinal muscle contractility in different rats, we used the baseline values of contractile tension and contractile frequency of the same rat to calculate the ratio of intestinal muscle contractile tension and contractile frequency at different concentrations of each drug, and then compared them.

### Statistical analysis

Data analysis was performed using SPSS 22.0 (SPSS, Inc., Chicago, IL, USA). The Kolmogorov‒Smirnov test was used to test the distribution of univariate data. The dose‒response curve was fitted with the logarithm of the drug concentration as the abscissa and the ratio of contraction tension and contraction frequency as the ordinate. Variables were expressed as medians (interquartile range), the Kruskal‒Wallis rank-sum test was used for comparisons between different concentrations of the same drug, and multiple comparisons were performed using the Dunnet statistical method. P < 0.05 was considered statistically significant.

## Results

**FIGURE 2:**
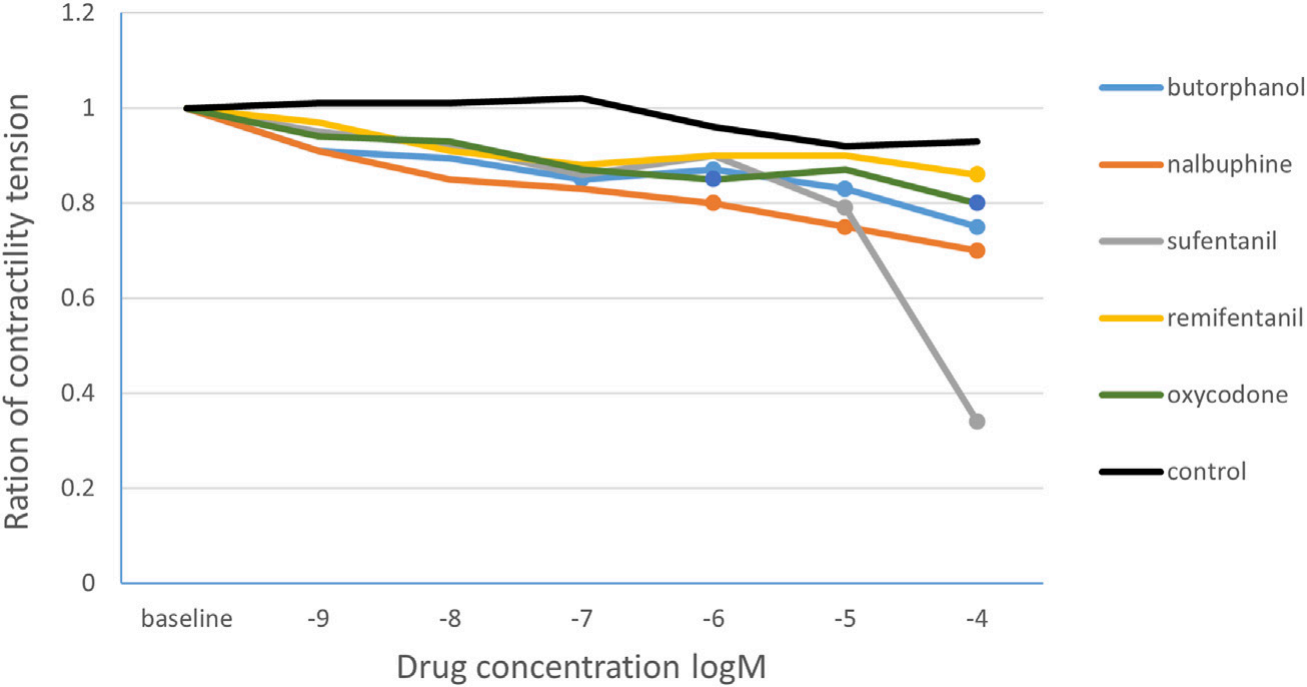
The change curve of the ratio of intestinal smooth muscle contraction tension under different concentrations of opioids. The solid circles are the drug concentrations corresponding to the statistically significant changes in the ratio of the contraction tension. Butorphanol 10^−7^ M, 10^−6^ M, 10^−5^ M,10^−4^ M concentrations; nalbuphine10^−6^ M, 10^−5^ M, 10^−4^ M concentration; oxycodone 10^−6^ M, 10^−4^ M concentration; sufentanil 10^−5^ M concentration; remifentanil 10^−4^ M concentration.

The data obtained from the *in vitro* intestinal tube experiment of 10 rats showed in Table 1. Statistical analysis found that butorphanol (10^−9^–10^−4^ M) decreased the tension ratio in a concentration-dependent manner. The first significant reduction occurred at 10^−7^ M (0.85 ± 0.26; p = 0.013 versus baseline). Significant inhibition persisted at 10^−6^, 10^−5^ and 10^−4^ M (p < 0.05). Nalbuphine produced its first significant effect at 10^−6^ M (0.80 ± 0.24; p = 0.027) and remained lower at 10^−5^ and 10^−4^ M (p < 0.05). Oxycodone significantly reduced tension at 10^−6^ M (0.85 ± 0.20; p = 0.024) and maintained inhibition up to 10^−4^ M (p < 0.05). Sufentanil and remifentanil decreased tension at 10^−5^ M (p = 0.010) and 10^−4^ M (p = 0.007), respectively. The control group showed no significant change, only a slight drift (Figure 2). Meanwhile, the tension ratio were compared with time-match control group, all differences remained significant except at 10^−6^ M butorphanol and nalbuphine produced significant changes at 10^−7^ M.

**TABLE 1:** The ratio of intestinal smooth muscle contraction tension with different concentrations of opioids.

Drug concentration	Butorphanol	Nalbuphine	Oxycodone	Sufentanil	Remifentanil	Control
10^−9^ M	0.91 (0.20)	0.91 (0.12)	0.94 (0.09)	0.95 (0.06)	0.97 (0.20)	1.01 (0.08)
10^−8^ M	0.89 (0.21)	0.85 (0.14)	0.93 (0.14)	0.92 (0.14)	0.91 (0.29)	1.01 (0.13)
10^−7^ M	0.85 (0.26)*^#^	0.83 (0.25)^#^	0.87 (0.19)	0.86 (0.18)	0.89 (0.27)	1.02 (0.13)
10^−6^ M	0.87 (0.20)*	0.80 (0.24)*^#^	0.85 (0.20)*^#^	0.90 (0.28)	0.90 (0.37)	0.96 (0.02)
10^−5^ M	0.83 (0.29)*^#^	0.75 (0.25)*^#^	0.87 (0.24)	0.79 (0.25)*^#^	0.90 (0.26)	0.92 (0.14)
10^−4^ M	0.75 (0.25)*^#^	0.70 (0.38)*^#^	0.80 (0.24)*^#^	0.34 (0.27)*^#^	0.86 (0.65)*	0.93 (0.16)

*Statistically significant difference from baseline P < 0.05.

# Statistically significant difference compared with time-match control group P < 0.05.

Data are presented as the median (interquartile range).

**FIGURE 3:**
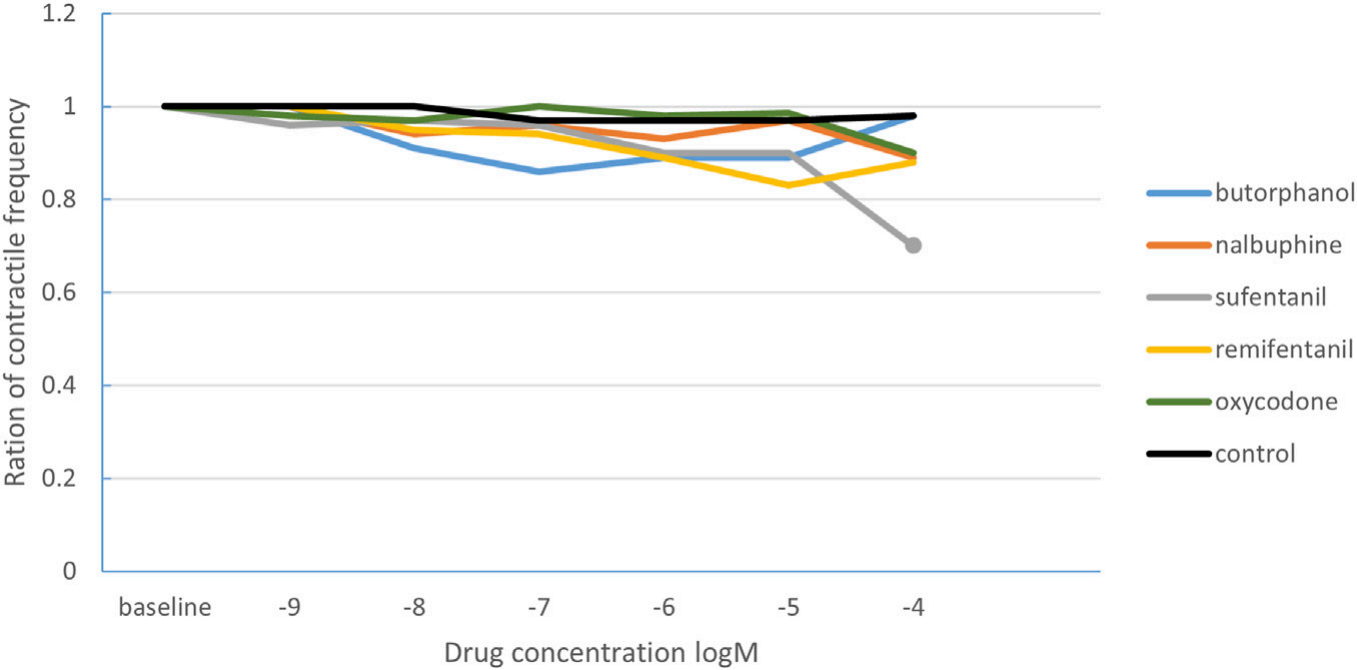
The change curve of intestinal smooth muscle contraction frequency ratio under different concentrations of opioids. The solid circles indicate the drug concentrations with statistically significant changes in the ratio of contraction frequency. The solid dots correspond to the sufentanil concentration of 10^−4^ M.

Regarding the effect on the contraction frequency of intestinal smooth muscle (Table 2), the ratio of contraction frequency decreased significantly in the sufentanil group when the drug concentration increased to 10^−4^ M compared with the baseline (0.7 [0.23], P < 0.001). There was no statistical difference among different concentrations of other drugs. In the control group, the contraction frequency remained virtually unchanged over time (Figure 3). After comparing the contraction frequency ratio with the time-matched control group, the statistical results were consistent.

**TABLE 2:** The ratio of intestinal smooth muscle contraction frequency of different concentrations of opioids.

Drug concentration	Butorphanol	Nalbuphine	Oxycodone	Sufentanil	Remifentanil	Control
10^−9^ M	1.00 (0.28)	1.00 (0.16)	0.98 (0.10)	0.96 (0.37)	1.00 (0.24)	1.00 (0.10)
10^−8^ M	0.91 (0.14)	0.94 (0.34)	0.97 (0.13)	0.97 (0.41)	0.95 (0.32)	1.00 (0.09)
10^−7^ M	0.86 (0.40)	0.96 (0.14)	1.00 (0.19)	0.96 (0.39)	0.94 (0.30)	0.93 (0.19)
10^−6^ M	0.89 (0.37)	0.93 (0.17)	0.98 (0.07)	0.9 (0.56)	0.89 (0.43)	0.97 (0.27)
10^−5^ M	0.89 (0.14)	0.97 (0.30)	0.95 (0.22)	0.9 (0.15)	0.83 (0.43)	0.97 (0.20)
10^−4^ M	0.98 (0.71)	0.89 (0.33)	0.9 (0.26)	0.7 (0.23)*	0.88 (0.48)	0.98 (0.19)

*Statistically significant change from baseline P < 0.05.

# Statistically significant difference compared with time-match control group P < 0.05.

Data are presented as the median (interquartile range).

## Discussion

In this study, by comparing the effects of commonly used opioid analgesics on the contraction tension and contraction frequency of the isolated intestinal canal at different concentrations, it was found that the inhibitory effect of opioids on isolated intestinal contraction was concentration dependent. The concentrations at which the intestinal contraction tension began to decrease significantly were butorphanol 10–^7^ M, nalbuphine and oxycodone 10–^6^ M, sufentanil 10–^5^ M, and remifentanil 10–^4^ M. According to the above results, the order of drugs for inhibiting intestinal contraction tension was as follows: butorphanol > nalbuphine ≈ oxycodone > sufentanil > remifentanil.

Opioids lead to postoperative gastrointestinal dysfunction by activating opioid receptors. The classic opioid receptors have three main receptor subtypes, MOP, KOP and DOP. Among the MOP receptor, MOP1 receptor are involved in analgesia and sedation; MOP2 receptor are involved in respiratory depression, pruritus, and gastrointestinal function inhibition; DOP receptors are involved in spinal cord analgesia, mediate smooth muscle effects and regulate MOP receptor activity (Hill and Canals, 2022; Galligan and Sternini, 2017; DiCello et al., 2020); and while the KOP receptor is involved in intestinal inhibition. Different research results have been reported. The KOP receptor agonist U50488H (1–100 mg/kg) was found to inhibit gastric emptying and small intestinal motility in male Wistar rats in a study of intraperitoneal injection (Asai et al., 1998), but the study on voluntary intestinal smooth muscle found that the KOP-receptor agonist U50488H had no effect on spontaneous contractile activity of muscle strips, which may partly account for the divergent results (Chamouard et al., 1993).

There are many types of opioids that we can choose clinically. The five drugs in the study are commonly used intraoperative and postoperative analgesics, and sufentanil and remifentanil are MOP receptor agonists (Gepts et al., 1995; Egan, 1998). Nalbuphine is a complete agonist of KOP receptors and a partial antagonist of MOP receptors (He et al., 2021); butorphanol mainly stimulates KOP receptors and has dual effects of agonism and antagonism on MOP receptors. The intensity of butorphanol on KOP∶MOP∶DOP receptors is 25∶4∶1,^10^ and oxycodone is a synthetic pure opioid receptor μ-k dual agonist (Raff et al., 2019). At present, no study has compared their effects on gastrointestinal function at the same time. Because of individual differences and drug limitations, it is difficult to study five drugs at the same time with postoperative gastrointestinal dysfunction as the main result. Therefore, we chose the rat intestinal tube for *in vitro* experiments for comparison. To minimize temporal bias, we used the same intestinal segment from each rat and included a time-matched control group, compared with time-match control group, the results remained virtually identical, indicating a weak time-dependent effect.

Of course, the effect of opioids on gastrointestinal function is a multichannel and complex process. In addition to directly acting on opioid receptors, it also inhibits the excitability of neurons in the enteric nervous system, resulting in the inhibition of the fluidity of intestinal contents. Drugs act on secretion-promoting neurons in the submucosal nerve plexus, inhibiting the release of neurotransmitters that induce secretory activity in the intestinal recess, reducing intestinal secretion, leading to decreased gastrointestinal function and the formation of constipation (Hughes et al., 2016; Kokki et al., 2012). While clinical research on opioids mainly focus on analgesic efficacy, postoperative gastrointestinal dysfunction often as incidental results (Sun et al., 2020; Han et al., 2018; Guo et al., 2022), which may be the reason why the results of clinical studies on the effects of opioids on gastrointestinal function differ from *in vitro* studies.

For this *in vitro* experiment, different molar concentrations of opioids calculated by molecular weight were selected for longitudinal comparison. Considering the clinical dose of each drug, the application of nalbuphine, butorphanol, and oxycodone is in mg for intraoperative and postoperative analgesia, while sufentanil and remifentanil are in μg. The blood concentrations of sufentanil and remifentanil at commonly used clinical doses are approximately 100–1,000 times lower than those of butorphanol and the other three drugs. Considering the equivalent analgesic dose between different drugs, we believe that the ranking of inhibition of these drugs on the intestinal smooth muscle will not change, and the difference will be more obvious. A previous *in vitro* study observed the effects of low, medium and high concentrations of morphine, sufentanil, and dezocine on ileal intestinal muscle contraction and further measured the changes of intestinal propulsion motion after intraperitoneal injection of three opioids in male Sprague-Dawley rats (Bian et al., 2015). While keeping the 20 mL bathing volume constant, the study showed that sufentanil at high doses (40–120 μg/L) concentration-dependently increased the tension of isolated rat small-intestinal smooth muscle, a finding that aligns with our results; dezocine, in contrast, had no significant effect on smooth-muscle contraction. Both opioids, however, may impair intestinal propulsive function. The results of Guo’s (Yan et al., 2021) research recently also supports our conjecture. The study investigated the effect of equivalent doses of fentanyl, oxycodone and butorphanol on small intestine segments function in patients undergoing laparoscopic hysterectomy and found that butorphanol significantly prolonged postoperative intestinal function recovery, followed by oxycodone. The first postoperative exhaust time of butorphanol, oxycodone and fentanyl was 45.2 ± 11.6, 36.2 ± 10.9 and 33.1 ± 11.2 h, respectively.

This study has several limitations. First, as mentioned above, our study chose molecular weight to calculate the unified experimental drug concentration, longitudinally compared the concentration gradient of opioids affecting gastrointestinal function, and made an indirect horizontal comparison based on the obtained results. Second, intestinal smooth muscle undergoes rhythmic contractions and it also performs circular contractions to complete the compound movement of the intestinal migration (Waterman et al., 1992), which may differ among segments, however we did not differentiate between intestinal segments. The effects of opioids on intestinal smooth muscle are multifaceted; in the present study we examined only changes in basal tension and did not explore potential non-opioid mechanisms or the influence of drugs on electrically or chemically evoked contractions. Finally, *in vitro* findings cannot be directly extrapolated to the *in vivo* situation; systemic neural and humoral inputs interact with the drug itself (Chen et al., 2012; Díaz-Ruano et al., 2019), and both opioid-receptor expression and intestinal physiology differ across species. The animal model offers a critical reference point for subsequent clinical work, but the study’s conclusions must await translational validation before they can be applied in practice.

## Conclusion

The study was found that different opioids affected the contractile tension of isolated rat intestinal smooth muscle with increasing drug concentration: butorphanol > nalbuphine ≈ oxycodone > sufentanil > remifentanil, and the contraction frequency was almost unaffected except in the very high-concentration sufentanil group. The consistency of this conclusion provides preliminary animal-model data on opioids, serving as a key reference for future *in-vivo* or clinical investigations aimed at guiding perioperative opioid selection with minimal gastrointestinal impact.

## Data Availability

The raw data supporting the conclusions of this article will be made available by the authors, without undue reservation.
